# EHMTI-0075. Is insomnia associated with migraineurs attributable to anxiety and depression?

**DOI:** 10.1186/1129-2377-15-S1-D10

**Published:** 2014-09-18

**Authors:** M Chu, SJ Cho, WJ Kim, JM Kim

**Affiliations:** 1 Neurology, Hallym University Sacred Heart Hospital, Anyang, Korea; 2 Neurology, Hallym University Sacred Heart Hospital, Hwaseong, Korea; 3 Neurology, Gangnam Severance Hospital Yonsei University College of Medicine, Seoul, Korea; 4 Neurology, Chungnam National University, Daejeon, Korea

## Introduction

The close relationship between insomnia and migraine has been documented in clinical and epidemiological studies. Anxiety and depression are often comorbid with migraine. Thus, it may be possible that insomnia in migraineurs is attributable to anxiety and depression.

## Aims

This study is to examine whether insomnia reported by migraineurs can be attributed to comorbid anxiety and/or depression.

## Methods

We selected a stratified random population sample of Koreans aged 19-69 and evaluated them with a 60-item semi-structured interview designed to identify headache type, anxiety, depression and insomnia. Anxiety and depression symptoms were evaluated using Goldberg Anxiety Scale questions and Patient Health Questionnnaire-9, respectively. We used Insomnia Severity Index (ISI) for diagnosis of insomnia (ISI score ≥15).

## Results

Of the 2,762 participants who completed the interview, 147 subjects (5.4%) were classified as having migraine during the previous year. One hundred twenty (4.3%) participants were diagnosed as having insomnia. Anxiety and depression were diagnosed in 274 (10.0%) and 124 (4.5%) participants, respectively. Among 147 subjects with migraine, 45 (30.1%) subjects had anxiety, 26 (17.7%) had depression and 15 (10.2%) had insomnia. Univariable logistic regression analysis revealed that migraine (OR [CI]) = 2.7 [1.5-4.7]), anxiety (OR [CI]) = 13.7 [9.3-20.1]) and depression (OR [CI]) = 27.6 [18.0-42.4]) showed significant associations with insomnia. After adjusting anxiety and depression, the association between migraine and insomnia disappeared (OR [CI]) = 0.8 [0.4-1.7]).

## Conclusion

Anxiety, depression and insomnia are common complaint of migraineurs. The association between migraine and insomnia was closely associated with comorbid anxiety and depression.

No conflict of interest.

